# The Effect of a Low-FODMAP Diet on Quality of Life, Severity of Symptoms and Leaky Gut in Patients with Irritable Bowel Syndrome

**DOI:** 10.3390/nu18142371

**Published:** 2026-07-20

**Authors:** Esra Demir, Yunus Can Ozalp, Hasan Basri Ergun, Burcu Uludag, Oktay Olmuşçelik, Gozde Ulfer, Omar Alomari

**Affiliations:** 1 Department of Internal Medicine, Istanbul Medipol University, 34810 Istanbul, Türkiye; yunuscanozalp@gmail.com (Y.C.O.); hbergun@gmail.com (H.B.E.); oolmuscelik@medipol.edu.tr (O.O.); 2 Department of Nutrition and Dietetics, Istanbul Medipol University, 34810 Istanbul, Türkiye; burcu.uludag@medipol.com.tr; 3 Department of Medical Biochemistry, Istanbul Medipol University, 34810 Istanbul, Türkiye; gozde.ulfer@medipol.com.tr; 4 Hamidiye International School of Medicine, University of Health Sciences, 34668 Istanbul, Türkiye; dromari2001@gmail.com

**Keywords:** Low-FODMAP diet, irritable bowel syndrome, leaky gut

## Abstract

**Objective**: We aimed to investigate the effects of a low-FODMAP (fermentable oligosaccharides, disaccharides, monosaccharides, and polyols) diet on symptom severity, quality of life, and fecal zonulin levels in patients with irritable bowel syndrome (IBS). **Methods**: Twenty-four patients with IBS were prospectively randomized to either a low-FODMAP diet (LFD) or a traditional diet (TD) for 4 weeks. IBS Symptom Severity Scale (IBS-SSS), IBS Quality of Life (IBS-QOL), and fecal zonulin levels were assessed at baseline and week 4 in both groups. In the LFD group, FODMAP-containing foods were gradually reintroduced after week 4, and all assessments were repeated at week 16. Changes in laboratory parameters, fecal zonulin levels, IBS-SSS, and IBS-QOL scores were evaluated. **Results**: Fecal zonulin levels did not differ significantly between groups at baseline or week 4, and no significant within-group changes were observed over time (all *p* > 0.05). IBS-SSS scores improved significantly at week 4 in both groups compared with the baseline (*p* < 0.05), with no significant difference in the magnitude of improvement between groups (*p* > 0.05). In the LFD group, IBS-SSS scores increased at week 16 compared with week 4 (*p* < 0.05). Similarly, IBS-QOL scores improved significantly at week 4 in both groups (*p* < 0.05), without significant between-group differences (*p* > 0.05). In the LFD group, IBS-QOL scores returned to baseline levels by week 16. **Conclusions**: Both the LFD and TD were associated with significant short-term improvements in IBS symptom severity and quality of life. However, the LFD did not demonstrate superiority over the TD. Furthermore, no statistically significant changes in fecal zonulin levels were observed in either group, although these findings should be interpreted cautiously. In the LFD group, some improvements observed at week 4 were attenuated following FODMAP reintroduction at week 16; however, these findings represent exploratory within-group observations and should not be interpreted as evidence of long-term treatment efficacy or sustained benefit following FODMAP reintroduction.

## 1. Introduction

IBS is a chronic functional bowel disorder marked by recurrent abdominal pain with altered bowel habits. The diagnosis is established according to the Rome IV criteria, which require recurrent abdominal pain associated with changes in bowel habits in the absence of an identifiable organic cause. There are four subtypes: IBS-D (diarrhea-predominant type), IBS-C (constipation-predominant type), IBS-M (mixed type), and IBS-U (undefined type) [1,2]. IBS has become an increasingly important cause of outpatient clinic visits, healthcare expenditures, and loss of productivity. According to 2021 meta-analysis, the worldwide prevalence rate is 10.1% (9.8–10.5%) in studies using the Rome 3 criteria and 3.8% (3.6–4.0%) in studies using the Rome 4 criteria. The studies showed that women had significantly higher rates of IBS than men, with an odds ratio of 1.7 (1.6–1.9) under Rome III criteria and 1.7 (1.5–1.9) under Rome IV criteria [3]. More recent studies have reported prevalence estimates of up to 15%, with annual healthcare expenditures exceeding USD 30 billion [4]. Because there is no definitive diagnostic test, complaints are of a subjective nature, and there is a lack of definitive treatment, there is international variation in diagnostic rates, which are often lower than anticipated [3]. Accordingly, the development of effective therapeutic strategies has become a major focus of current research. From a pathophysiological perspective, it is evident that certain dietary components and disruptions in gut permeability contribute to the pathogenesis of IBS [5,6,7].

Zonulin is a signaling protein primarily secreted by the intestinal epithelium, although it is also expressed in several extraintestinal tissues, including the liver, lungs, kidneys, brain, and heart. By modulating the intercellular tight junctions (TJs) between the intestinal lumen and the interstitium, zonulin regulates intestinal barrier permeability. Therefore, fecal zonulin has been proposed as a non-invasive biomarker of intestinal permeability and can be used to evaluate the effects of an LFD on gut barrier function in patients with IBS [8]. TJs are composed of transmembrane proteins—including occludin, claudins, and junctional adhesion molecules (JAMs)—as well as cytoplasmic scaffold proteins such as ZO-1, ZO-2, ZO-3, cingulin, and 7H6 [9]. Disruptions in gut microbiota composition can trigger the upregulation of zonulin expression. This increased zonulin activity leads to the loosening of TJs, resulting in compromised intestinal barrier function, commonly referred to as leaky gut. Consequently, macromolecules, antigens, and immune cells such as leukocytes—normally restricted from systemic entry—can cross the now-permeable epithelial barrier. This phenomenon has been implicated in the pathogenesis of various autoimmune, allergic, infectious, and neoplastic diseases [5,9,10].

Diets rich in fructo-oligosaccharides and fructose—key components of FODMAPs—have been shown in animal studies to promote the proliferation of Gram-negative bacteria in the gut microbiota. Lipopolysaccharides (LPS), located in the outer membrane of Gram-negative bacteria, can compromise intestinal barrier function by activating toll-like receptor 4 (TLR-4) on mast cells, triggering the release of inflammatory mediators such as IL-6, IL-1β, TNF-α, and proteases [11]. High-FODMAP diets may increase intestinal permeability by disrupting tight-junction integrity, thereby contributing to the development and exacerbation of IBS symptoms. In contrast, low-FODMAP diets have been shown to improve symptoms and enhance quality of life in IBS patients, possibly by improving intestinal barrier integrity [12]. Not only FODMAPs but also several dietary factors have been shown to adversely affect gut health. Red meat, processed meat, foods rich in trans fatty acids, refined sugars, an excess of *n*-6 polyunsaturated fatty acids, and saturated fats may reduce microbial α-diversity and promote the overgrowth of pathobionts. Conversely, some foods containing FODMAPs, such as fiber-rich vegetables and fruits, may exert beneficial effects by increasing microbial diversity, enhancing the abundance of beneficial bacteria, and reducing potentially harmful species. Therefore, while an LFD has proven efficacy in improving IBS-related symptoms, long-term strict adherence may negatively impact microbial diversity. For this reason, once symptom control has been achieved, it is important to reintroduce as many foods as tolerated in order to maintain or restore the gut microbiota. The appropriate indications, duration, and limitations of an LFD are still not fully defined, and a balanced approach to reintroduction is recommended [13].

IBS symptom severity and quality of life were assessed using the IBS Symptom Severity Scale (IBS-SSS) and the IBS Quality of Life (IBS-QOL) questionnaire, allowing for objective evaluation before and after the dietary intervention [12,14,15,16]. In the present study, fecal zonulin levels were measured to evaluate the effects of the LFD on intestinal barrier function in patients with IBS.

This study aimed to evaluate the short-term effects of a low-FODMAP diet on intestinal permeability, symptom severity, and quality of life in patients with IBS. Additionally, an exploratory follow-up was conducted in the LFD group after gradual reintroduction of FODMAP-containing foods to assess within-group changes after completion of the elimination phase of the diet.

## 2. Materials and Methods

This study was designed as a randomized, single-blind, prospective study. The study was conducted at Istanbul Medipol Mega University Hospital. Patients were recruited between January and February 2026. Follow-up assessments were completed in May 2026, and data verification and statistical analyses were finalized in June 2026.

A total of 37 patients aged between 18 and 65 years, who presented to the Internal Medicine and Gastroenterology outpatient clinics, were enrolled in the study. The diagnosis of IBS was established according to the Rome IV criteria [2]. Patients were excluded if they had received IBS-specific treatment within the previous month; used antibiotics or probiotics within the previous two weeks; had a history of major abdominal surgery; or had active malignancy, heart failure, renal failure, autoimmune disease, inflammatory bowel disease, celiac disease, or lactose intolerance. Individuals who were currently following any dietary regimen or pregnant women were also excluded. Our study was approved by the Istanbul Medipol University Non-Interventional Clinical Research Ethics Committee (ethics committee decision number: E-10840098-772.02-5814). This study was registered at ClinicalTrials.gov (identifier: NCT07467278 (Registration date: 12 March 2026)). Participants were included irrespective of IBS subtype.

Eligible patients were randomized into LFD and TD groups using computer-generated randomization, with a ratio of 1:1. Sealed opaque envelopes were used, and the assigned dietary intervention was provided by an expert dietitian. The study was conducted using a single-blind design in which participants were unaware of their treatment allocation. Patients were informed only that they would receive a dietary intervention for IBS but were not told whether they had been assigned to the low-FODMAP diet or the traditional diet. Dietary counseling for both groups was delivered by the same dietitian using standardized procedures to minimize disclosure of group allocation. The participants submitted their dietary diaries weekly to the dietitian via telephone. Dietary compliance was assessed using participants’ dietary diaries by expert clinicians using a standardized study questionnaire. Compliance was categorized as follows: non-compliant (<25%), occasionally compliant (25–50%), mostly compliant (51–75%), and fully compliant (>76%). (15). Only patients who were mostly or fully compliant with the diet were included in the study.

Demographic and clinical data, including age, body mass index, comorbidities, medication use, IBS-SSS, and IBS-QOL scores, were recorded at baseline, after completion of the dietary intervention, and at long-term follow-up.

The IBS-SSS is a validated tool based on a visual analog scale (VAS), comprising five subscores that assess the severity of IBS-related symptoms over the preceding 10 days. These subscores evaluate the intensity and duration of abdominal pain, abdominal bloating, stool frequency and consistency, and the overall impact of symptoms on daily life. Each subscore yields a maximum of 100 points, resulting in a total score ranging from 0 to 500. Scores <75 indicate remission, 75–175 represent mild disease, 175–300 indicate moderate disease, and >300 represent severe disease. Clinical improvement was defined as a reduction of at least 50 points in the total IBS-SSS score or a decrease of ≥10 points in any of the five individual subscores during the study period [12,17].

The IBS-QOL score assesses the impact of IBS-related complaints on patients’ quality of life over the preceding month. It is derived from a 34-item questionnaire, each rated on a 5-point Likert scale: 1 = no problem; 2 = mild (negligible, not affecting daily activities); 3 = moderate (not negligible, but not interfering with daily activities); 4 = severe (affecting concentration during daily activities); and 5 = very severe (significantly interfering with daily activities and/or requiring rest). The total score ranges from 34 to 170. For the IBS-QOL questionnaire, higher scores indicate poorer quality of life; therefore, a reduction in score reflects improvement [18].

Fresh stool samples were collected from all participants and stored at −80 °C within 12 h of collection. At the end of the study, the samples were thawed and analyzed using a Biotek Synergy HTX multi-mode reader (BioTek Instruments, Winooski, VT, USA) together with a stool sample preparation kit (Immundiagnostik AG, Bensheim, Germany). Fecal zonulin concentrations were measured according to the manufacturer’s instructions.

### 2.1. Visit-1

At the beginning of the dietary intervention, stool samples were collected from all participants for the measurement of fecal zonulin levels, and both the IBS-SSS and IBS-QOL questionnaires were administered. A registered dietitian provided individualized dietary instructions to each patient, including detailed explanations of permitted and prohibited foods, as well as food exchange lists. Participants in the control group received a traditional diet based on commonly consumed foods in the Turkish diet and consistent with the principles of healthy eating. Participants were encouraged to consume regular meals including cereals and bread, rice, bulgur, vegetables, fruits, dairy products (milk, yogurt, and cheese), legumes, eggs, poultry, fish, and lean meat. Olive oil was recommended as the primary dietary fat. Participants were advised to limit foods that may exacerbate gastrointestinal symptoms, including excessive caffeine-containing beverages, alcohol, high-fat fried foods, processed foods rich in refined sugars, and highly spicy foods. No restrictions were imposed on FODMAP-containing foods.

Dietary adherence was monitored through weekly telephone consultations, during which patients’ food diaries were reviewed by the dietitian. Adherence was determined by calculating the proportion of meals that complied with the prescribed dietary recommendations. Only participants with an adherence rate of ≥51% were included in the final analysis. Patients were excluded if they demonstrated insufficient adherence to the diet, failed to submit their food diaries consistently, or required antibiotic treatment during the intervention period. Ultimately, 24 patients successfully completed the dietary intervention and were included in the final analysis.

### 2.2. Visit-2

Following 4 weeks of dietary intervention, the participants in both groups were evaluated. Stool samples were collected for fecal zonulin measurement, and the IBS-SSS and IBS-QOL questionnaires were re-administered.

### 2.3. Visit-3

Following the week 4 stool sample collection and questionnaire assessments in the LFD group, a structured food reintroduction protocol was initiated. Over the subsequent 12 weeks, previously restricted high-FODMAP foods were gradually reintroduced, with two to three new food items added each week. At the end of week 16, stool samples were collected for the measurement of fecal zonulin levels, and the IBS-SSS and IBS-QOL questionnaires were administered to evaluate changes following the food reintroduction phase.

Only participants in the LFD group underwent follow-up assessment at week 16. Participants in the Traditional Diet group completed the study protocol after the week 4 assessment and did not undergo further follow-up. Consequently, all week 16 analyses were conducted exclusively within the LFD group, and no between-group comparisons were performed at this time point.

### 2.4. Statistical Analysis

The distribution of the study variables was assessed separately within each group by evaluating skewness and kurtosis coefficients. Variables with skewness and kurtosis values between −2 and +2 were considered to satisfy the assumption of approximate normality.

As part of the preliminary analyses, baseline comparability between the study groups was assessed before evaluating changes in outcome measures over time and between groups. For categorical variables, Fisher’s exact test was used when at least one expected cell count was less than 5. Between-group comparisons were performed using the independent-sample *t*-test for variables that met the assumption of normality and the Mann–Whitney U test for variables that did not meet the normality assumption.

Because participants who discontinued the intervention due to non-adherence did not complete the week 4 assessment and therefore had no post-intervention outcome data, an intention-to-treat analysis was not feasible. Consequently, all analyses were performed on a per-protocol basis, including only participants who completed the dietary intervention and all scheduled outcome assessments.

To compare changes in outcome measures over time between the study groups, linear mixed-effect models were applied using data collected at baseline and week 4. Group, time, and the group × time interaction were included as fixed effects. Baseline values and IBS subtype (IBS-C vs. non-IBS-C) were entered as covariates to account for baseline imbalance and differences in IBS subtype distribution. The within-subject correlation of repeated measurements was modeled using a compound symmetry covariance structure, and model parameters were estimated using restricted maximum likelihood (REML). Adjusted group means (estimated marginal means) were calculated, and pairwise comparisons were performed using the Bonferroni adjustment. The normality of model residuals was assessed by examining skewness and kurtosis coefficients. Effect sizes for the fixed effects were reported as partial eta squared (ηp^2^). Because only participants in the LFD group were evaluated at week 16, these analyses were performed separately within the LFD group and were considered exploratory.

Statistical analyses were performed using IBM SPSS Statistics (version 28). Data visualization was conducted in the R version 4.5.1. Data processing and management were performed using the dplyr and tidyr packages, whereas figures were generated using the ggplot2 and cowplot packages. A two-sided *p* value < 0.05 was considered statistically significant for all analyses. A post hoc power analysis was performed for the between-group comparisons of change scores in the primary outcomes, including fecal zonulin, IBS-SSS, and IBS-QOL. The analysis was conducted using a two-sided independent-sample *t*-test framework, with α = 0.05 and equal group sizes of 12 participants per group. Power was calculated based on the observed effect sizes for the change scores from baseline to week 4.

Generative artificial intelligence tools were used solely to improve the English language and readability of the manuscript. All scientific content, study design, statistical analyses, interpretation of the results, and final approval of the manuscript were performed exclusively by the authors.

## 3. Results

**Figure 1:**
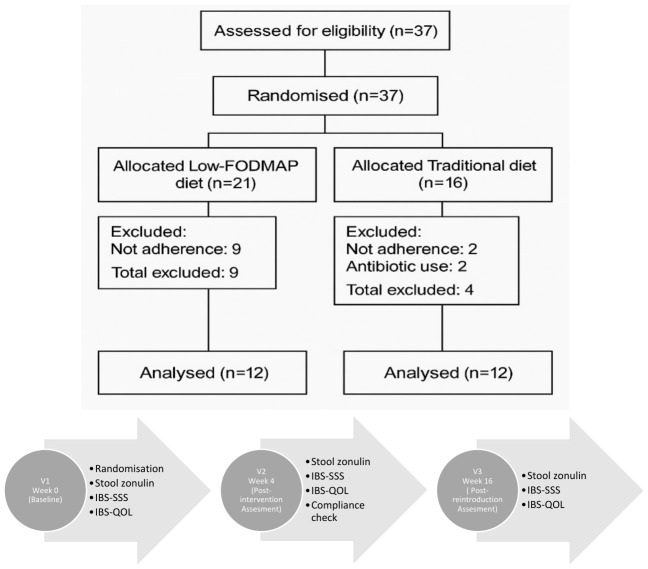
Consort diagram and study timeline.

A total of 37 patients were initially enrolled in the study. During the intervention, nine patients in the LFD group and two in the Traditional Diet group were excluded because of insufficient dietary adherence. An additional two patients were excluded due to antibiotic use during the intervention period. Ultimately, 24 patients (12 in the LFD group and 12 in the Traditional Diet group) completed the study protocol and were included in the final per-protocol analysis (Figure 1).

Table 1 presents the baseline sociodemographic, clinical, and biochemical characteristics of the Traditional Diet and Low-FODMAP Diet (LFD) groups. No statistically significant between-group differences were observed in age, sex, body mass index (BMI), IBS subtype distribution, white blood cell count (WBC), hemoglobin (Hgb), platelet count (Plt), alanine aminotransferase (ALT), aspartate aminotransferase (AST), creatinine, or C-reactive protein (CRP) (all *p* > 0.05).

Table 2 presents the results of the linear mixed-effect models for zonulin, IBS Symptom Severity Score (IBS-SSS), and IBS Quality of Life (IBS-QOL), adjusted for baseline values and IBS subtype, in the Low-FODMAP Diet (LFD) and Traditional Diet groups. After adjustment for baseline imbalances, no statistically significant between-group differences were observed in the adjusted week 4 means. The group effect was not significant for zonulin (*p* > 0.05), and no significant group effects were detected for IBS-SSS or IBS-QOL (*p* > 0.05). These findings indicate that, after accounting for baseline differences, the two dietary interventions yielded comparable outcomes at week 4.

With respect to the effect of time, the change in IBS-QOL from week 4 to week 16 was of borderline statistical significance (*p* = 0.050), whereas no significant changes over time were observed for zonulin or IBS-SSS (*p* > 0.05). Among the covariates, baseline values were significant predictors of zonulin (*p* < 0.001) and IBS-QOL (*p* < 0.05). In contrast, IBS subtype was significantly associated only with IBS-SSS (*p* < 0.05).

Table 3 presents the comparisons of baseline, week 4, and change scores for zonulin, IBS Symptom Severity Score (IBS-SSS), and IBS Quality of Life (IBS-QOL) between the Low-FODMAP Diet (LFD) and Traditional Diet groups. No statistically significant between-group differences were observed in baseline values, week 4 measurements, or change scores for zonulin (all *p* > 0.05).

For IBS-SSS, a statistically significant between-group difference was found at baseline (*p* < 0.05), with the Traditional Diet group exhibiting higher baseline scores than the LFD group. However, no significant between-group differences were found in week 4 scores or change scores (*p* > 0.05).

Post hoc power analysis showed that the achieved statistical power for detecting between-group differences in change scores was 42.2% for fecal zonulin, 22.0% for IBS-SSS, and 5.0% for IBS-QOL.

**Figure 2:**
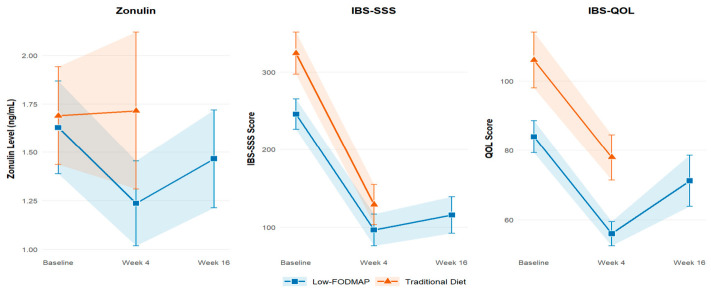
Temporal changes in zonulin, IBS-SSS, and IBS-QOL in the Traditional Diet and Low-FODMAP Diet groups. *Higher IBS-QOL scores indicate poorer quality of life*. Note. IBS-SSS (Irritable Bowel Syndrome–Symptom Severity Score); IBS-QOL (Irritable Bowel Syndrome–Quality of Life).

For IBS-QOL, statistically significant between-group differences were observed at both baseline and week 4 (*p* < 0.05), with the Traditional Diet group demonstrating higher scores than the LFD group at both time points. In contrast, the change in IBS-QOL from baseline to week 4 did not differ significantly between the two groups (*p* > 0.05). The temporal changes in each outcome measure for both groups are illustrated in Figure 2.

## 4. Discussion

The present study adds to the limited body of prospective randomized controlled trials investigating the effects of a low-FODMAP diet on symptom severity, health-related quality of life, and fecal zonulin concentrations in patients with IBS. Our findings indicate that both dietary approaches were associated with significant improvements in symptom severity and health-related quality of life during the intervention period. Although no statistically significant between-group differences were observed, both dietary interventions produced reductions in IBS-SSS scores that exceeded the commonly accepted minimum clinically important difference (MCID) of 50 points, indicating that the observed within-group improvements were clinically meaningful despite the absence of between-group superiority. While the LFD group exhibited significant within-group clinical improvements, these changes were not accompanied by a statistically significant reduction in fecal zonulin levels. Accordingly, present findings do not support a clear association between symptom improvement and changes in fecal zonulin concentrations in this cohort.

However, these findings should be interpreted with caution, as baseline IBS-SSS scores differed between groups and IBS-D was numerically more prevalent in the LFD group, although the overall distribution of IBS subtypes did not differ significantly. These baseline characteristics may have influenced symptom severity and treatment response. FODMAPs are osmotically active compounds that increase luminal water content and lead to enhanced gas production as a result of fermentation by the colonic microbiota. Retrospective studies have demonstrated that restricting these fermentable carbohydrates from the diet may result in a substantial improvement in IBS symptoms [6]. Consistent with these findings, our study demonstrated a significant reduction in IBS-SSS scores over time in both the Traditional Diet and LFD groups. Notably, within-group analyses revealed marked improvements in the LFD group, supporting previous evidence that a low-FODMAP diet may be effective in reducing gastrointestinal symptoms in patients with IBS [6,13,18,19].

Similarly, significant improvements in IBS-QOL scores were observed in the LFD group, in agreement with previous reports [13,18]. However, these improvements were no longer evident at week 16 following the structured food reintroduction phase. Within the LFD group, this finding may suggest a partial loss of the improvements observed during the elimination phase. However, because week-16 assessments were performed only in the LFD group, these findings should be interpreted as exploratory hypothesis-generating within-group observations rather than controlled evidence regarding the long-term efficacy of the low-FODMAP diet. Further studies with long-term follow-up in both intervention groups are needed to clarify the durability of dietary effects.

Several studies have reported that both standard and modified low-FODMAP dietary interventions may provide sustained benefits beyond placebo effects, leading to improvements in IBS symptoms, psychological well-being, and quality of life [20]. Furthermore, low-FODMAP dietary approaches have been shown to exert favorable effects on symptom burden, particularly in patients with refractory disease and functional dyspepsia coexisting with IBS [21]. Currently, the low-FODMAP diet has become an established therapeutic strategy in the management of IBS, and accumulating evidence suggests that it may also confer benefits in other disorders involving the gut–brain axis as well as in chronic pain conditions. In addition, several studies support the long-term efficacy of modified low-FODMAP diets in improving both IBS-related symptoms and anxiety–depressive manifestations. The presence of fibromyalgia has not been reported to have a significant adverse impact on either global symptom response or adherence to dietary intervention [22].

The observed decline in quality of life after diet discontinuation also raises concerns that prolonged FODMAP restriction may contribute to potential nutritional deficiencies. While there are some studies showing that short-term LFD administration does not cause nutritional deficiency [23], there is a lack of evidence in the current literature regarding the nutritional outcomes of long-term adherence to this dietary approach. The elimination phase of the LFD is not designed for long-term use. Therefore, it is important to identify trigger foods and reintroduce appropriate nutrients into the diet under the guidance of a dietitian. This approach may prevent nutritional deficiencies. The impact of various dietary patterns on the gut microbiota has been explored in the literature, and Mediterranean-type diets have been shown to exert beneficial effects on intestinal permeability similar with LFD [24].

Tests commonly used to evaluate gastrointestinal barrier function—particularly TJ integrity—include measurements of fecal and serum zonulin levels. Zonulin is a human protein that reversibly modulates intestinal permeability by regulating intercellular TJs. Additionally, intestinal fatty acid-binding protein (I-FABP) and diamine oxidase (DAO) are considered potential biomarkers of intestinal epithelial barrier health, as both are rapidly released into the circulation in response to compromised membrane integrity [25].

The findings related to fecal zonulin levels, a biomarker commonly associated with intestinal permeability (“leaky gut”), should be interpreted with caution. Commercially available ELISA kits for zonulin, including the assay used in this study, have been reported to detect not only zonulin (prehaptoglobin-2) but also structurally related proteins such as properdin. Consequently, the term *zonulin family proteins* (ZFPs) has been proposed to more accurately describe these measurements, and concerns have been raised regarding the specificity and clinical interpretability of these assays, particularly in fecal samples [26,27,28]. Although fecal ZFP levels decreased numerically following the low-FODMAP diet, the changes did not reach statistical significance. This finding may reflect the limited specificity of currently available fecal assays, the relatively small sample size, and limited statistical power of the present study, or a true absence of an effect on intestinal permeability. Likewise, previous studies have suggested that a low-FODMAP diet has limited or no measurable effect on gut microbiome diversity [29]. It is also possible that changes in intestinal barrier function may have been detected more accurately using validated serum biomarkers, such as serum zonulin, intestinal fatty acid-binding protein (I-FABP), or diamine oxidase (DAO), as reported in previous studies [18]. Therefore, our findings should not be interpreted as definitive evidence that a low-FODMAP diet has no effect on intestinal barrier function but rather as inconclusive evidence within the methodological limitations of currently available fecal zonulin assays.

Some studies suggest that zonulin levels may be influenced not only by intestinal permeability but also by alterations in gut microbiota composition. FODMAP restriction, while reducing fermentable substrates, may also decrease the abundance of beneficial bacteria such as *Bifidobacteria*, potentially counteracting the expected decline in zonulin levels [6]. Accordingly, the findings of the present study suggest that although a low-FODMAP diet may improve IBS symptoms and quality of life, its effects on intestinal barrier function remain uncertain. The absence of a significant change in fecal zonulin levels may reflect the multifactorial regulation of intestinal permeability, the limitations of currently available fecal zonulin assays, or an effect that could not be detected using the present methodology. Future studies with larger sample sizes, longer follow-up, and more comprehensive assessments incorporating validated biomarkers of intestinal permeability and mucosal injury, such as serum zonulin, intestinal fatty acid-binding protein (I-FABP), diamine oxidase (DAO), and alpha-1-antitrypsin, are needed to better clarify the mechanisms underlying the clinical benefits of a low-FODMAP diet.

## 5. Conclusions

In this study, patients with IBS who followed a low-FODMAP diet experienced improvements in symptom severity and quality of life. However, similar improvements were also observed in the Traditional Diet group, suggesting that the superiority of the low-FODMAP diet should be interpreted with caution. Furthermore, within the LFD group, some of the improvements observed during the elimination phase appeared to diminish following dietary reintroduction. However, because long-term follow-up data were not available for the Traditional Diet group, these findings should be considered exploratory, hypothesis-generating within-group observations rather than controlled evidence regarding the long-term efficacy of the low-FODMAP diet.

No significant changes in fecal zonulin levels were observed in our study. These findings suggest that the effects of a low-FODMAP diet on intestinal permeability cannot be clearly demonstrated. The absence of statistically significant changes should not be interpreted as evidence of the absence of a biological effect, as the study may have been underpowered, and fecal zonulin measurement is subject to important methodological limitations. Further large-scale studies incorporating additional validated biomarkers of intestinal permeability are warranted to better elucidate the relationship between dietary interventions, intestinal barrier function, and clinical outcomes in patients with IBS.

### Limitations

The principal limitation of this study is the relatively small sample size, which may have reduced the statistical power to detect small-to-moderate between-group differences in fecal zonulin levels, IBS-SSS, and IBS-QOL outcomes. Consistent with this limitation, the post hoc power analysis demonstrated low achieved power for the primary outcomes, particularly for IBS-SSS and IBS-QOL. Therefore, the non-significant between-group findings should be interpreted as inconclusive rather than definitive evidence of no difference between dietary interventions. Consequently, the absence of statistically significant differences should not be interpreted as definitive evidence of no effect, and the possibility of a Type II error cannot be excluded. Another important limitation is the relatively high attrition rate in the LFD group. Because the analyses were performed on a per-protocol basis, participants who successfully completed the dietary intervention may have differed systematically from those who discontinued follow-up, introducing potential selection bias and limiting the generalizability of the findings. Moreover, the high attrition rate may also reflect the practical challenges of implementing and maintaining a low-FODMAP diet in routine clinical practice.

Participants were enrolled without stratification according to IBS subtype. Although no statistically significant between-group differences were observed in IBS subtype distribution, the LFD group contained a greater numerical proportion of patients with IBS-D. Given that IBS-D has been associated with increased intestinal permeability and altered zonulin levels, a potential influence of this distribution on the study outcomes cannot be completely excluded. In addition, although the difference was not statistically significant, the unequal sex distribution between groups, with no male participants in the Traditional Diet group, may further limit the generalizability of the findings.

Finally, intestinal barrier function was assessed solely by fecal zonulin measurement. The absence of concurrent evaluation using additional validated biomarkers, such as serum zonulin, intestinal fatty acid-binding protein (I-FABP), and diamine oxidase (DAO), limited the comprehensiveness of the assessment. Furthermore, commercially available fecal zonulin ELISA assays have recognized limitations in specificity and may detect structurally related proteins rather than zonulin alone. Therefore, the absence of significant changes in fecal zonulin levels should be interpreted cautiously and may reflect methodological limitations of the assay rather than the true absence of a biological effect.

## Figures and Tables

**Table 1:** Comparison of baseline sociodemographic, clinical, and biochemical characteristics between the Traditional Diet and Low-FODMAP Diet groups.

Variables	Traditional Diet Group (*n:*12)		LFD Group		Test Variable	*p*	Effect Size
			(*n:*12)				
	Mean ± sd/n-%	Median/IQR	Mean ± sd/n-%	Median/IQR			
Age	36.45 ± 10.38	34.00/14.00	33.73 ± 10.57	32.00/12.00	0.983	0.326 *^z^*	0.201
Gender							
*Female*	12 (100.0%)		9 (75.0%)		-	0.217 *^f^*	0.378
*Male*	0 (0.0%)		3 (25.0%)				
BMI	23.44 ± 2.85	24.00/3.80	22.90 ± 3.43	22.49/4.24	−0.558	0.582 *^t^*	0.228
IBS subgroup							
*C*	10 (83.3%)		7 (58.3%)		-	0.128 *^f^*	0.450
*D*	0 (0.0%)		4 (33.3%)				
*M*	2 (16.7%)		1 (8.3%)				
Wbc	7.14 ± 1.78	7.11/2.61	6.56 ± 1.93	6.34/3.11	−0.517	0.611 *^t^*	0.211
Hgb	12.65 ± 1.07	12.60/1.30	13.71 ± 1.47	13.40/2.60	1.854	0.077 *^t^*	0.757
Plt	226.45 ± 51.49	213.00/46.00	259.82 ± 71.52	261.00/67.00	1.602	0.123 *^t^*	0.654
Alt	14.45 ± 4.28	14.00/7.00	15.64 ± 8.68	12.00/10.00	0.342	0.737 *^t^*	0.14
Ast	16.00 ± 5.81	14.00/6.00	16.18 ± 3.43	16.00/5.00	0.043	0.966 *^t^*	0.018
Creatinin	0.70 ± 0.10	0.68/0.13	0.70 ± 0.12	0.70/0.17	−0.244	0.810 *^t^*	0.100
Crp	1.35 ± 1.33	0.56/2.45	1.92 ± 2.45	1.56/1.47	−0.616	0.538 *^z^*	0.126

SD, standard deviation; IQR, interquartile range; *z*, Mann–Whitney *U* test; *f*, Fisher’s exact test; *t*, independent-sample *t*-test. Effect sizes were reported as *r* (*r* = *z*/√*N*) for the Mann–Whitney *U* test, Phi (φ) for Fisher’s exact test in 2 × 2 contingency tables, Cramér’s *V* for Fisher’s exact test in 2 × 3 contingency tables, and Hedges’s *g* for the independent-sample *t*-test.

**Table 2:** Linear mixed-effect model results for zonulin, IBS-SSS, and IBS-QOL adjusted for baseline values and IBS subtype in the Low-FODMAP Diet and Traditional Diet groups.

Measurement	Effect/Time	LFD	Traditional Diet	Difference(LFD–TDG)	F	*p*	ηp^2^
		Adj. Mean ± SH	Adj. Mean ± SH	(95% CI)			
IBS-SSS	Week 4 (T1)	96.161 ± 21.222	130.178 ± 23.471				
	Week 16 (T2)	115.328 ± 21.222	— ^b^				
	Group effect			−34.016 (−103.839, 35.806)	1.019	0.324	0.044
	Time effect				1.918	0.193	0.144
	Baseline (cov.)				1.976	0.174	0.086
	IBS subgroup (cov.)				7.282	0.014	0.270
IBS-QOL	Week 4 (T1)	59.168 ± 5.608	71.580 ± 6.271				
	Week 16 (T2)	74.335 ± 5.608	— ^b^				
	Group effect ^a^			−12.411 (−30.844, 6.021)	1.895	0.179	0.061
	Time effect				4.585	0.050	0.239
	Baseline (cov.)				7.544	0.011	0.226
	IBS subgroup (cov.)				0.247	0.625	0.013
Zonulin	Week 4 (T1)	1.276 ± 0.230	1.634 ± 0.236				
	Week 16 (T2)	1.506 ± 0.230	—^b^				
	Group effect ^a^			−0.357 (−1.054, 0.339)	1.137	0.298	0.051
	Time effect				2.487	0.145	0.197
	Baseline (cov.)				24.969	<0.001	0.569
	IBS subgroup (cov.)				0.485	0.495	0.026

Adj. Mean, adjusted mean (estimated marginal mean); values are presented as adjusted mean ± standard error (SE). TDG, Traditional Diet group; CI, confidence interval; ηp^2^, partial eta squared; cov., covariate. The linear mixed-effect model was fitted using restricted maximum likelihood (REML) with a compound symmetry covariance structure. Covariates were held constant at the following values: baseline IBS-SSS = 271.94, baseline IBS-QOL = 91.28, baseline zonulin = 1.648, and IBS subtype = 0.667. Partial eta squared (ηp^2^) values were calculated from the corresponding *F* statistics and degrees of freedom. **^a^** *F*, *p*, and ηp^2^ values represent the adjusted main effect of group (between-group comparison) after controlling for baseline values and IBS subtype. Because no week 16 measurements were available for the Traditional Diet group, the group × time interaction could not be estimated. Consequently, the between-group comparison is presented only for the estimable adjusted mean at week 4. **^b^** The adjusted mean for week 16 in the Traditional Diet group was not estimable because no measurements were available at this time point. Because week 16 measurements were not available for the Traditional Diet group, the adjusted mean for this group at week 16 could not be estimated, precluding estimation of the group × time interaction. Therefore, the week 16 findings should be interpreted as within-group results for the Low-FODMAP Diet group rather than as between-group comparisons.

**Table 3:** Comparison of baseline, week 4, and change scores of zonulin, IBS-SSS, and IBS-QOL between the Low-FODMAP Diet and Traditional Diet groups.

Measurement		LFD Group (*n*:12)		Traditional Diet Group (*n*:12)		Test Statistic	*p*	Effect Size
		Mean ± sd	Median/IQR	Mean ± sd	Median/IQR			
Zonulin	Baseline (T0)	1.63 ± 0.83	1.40/1.75	1.69 ± 0.88	1.76/1.13	0.115	0.908 *^Z^*	0.020
	Week 4 (T1)	1.24 ± 0.75	1.17/1.15	1.71 ± 1.40	1.47/1.20	0.866	0.386 *^Z^*	0.180
	*Difference (T1-T0)*	−0.39 ± 0.73	−0.49/1	0.03 ± 0.87	−0.22/1.03	−1.277	0.215 *^t^*	0.500
IBS-SSS	Baseline (T0)	245.83 ± 67.35	240.00/123.00	324.17 ± 93.66	345.00/140.00	−2.352	0.028 *^t^*	0.930
	Week 4 (T1)	96.67 ± 70.24	75.00/128.00	129.17 ± 89.08	120.00/163.00	−0.992	0.332 *^t^*	0.390
	*Difference (T1-T0)*	−149.17 ± 74.40	−160/92.50	−195.00 ± 104.32	−205/145	1.239	0.228 *^t^*	0.490
IBS-QOL	Baseline (T0)	83.92 ± 16.05	80.50/24.00	106.00 ± 27.81	108.00/31.00	−2.383	0.026 *^t^*	0.940
	Week 4 (T1)	56.00 ± 11.99	57.00/23.00	77.92 ± 22.50	73.00/44.00	−2.978	0.007 *^t^*	1.170
	*Difference (T1-T0)*	−27.92 ± 20.51	−26/36.25	−28.08 ± 17.48	−29.50/34	0.021	0.983 *^t^*	0.010

IBS-SSS, Irritable Bowel Syndrome Symptom Severity Score; IBS-QOL, Irritable Bowel Syndrome Quality of Life; T0, baseline assessment; T1, week 4 assessment; SD, standard deviation; IQR, interquartile range; *Z*, Mann–Whitney U test; *t*, independent-sample *t*-test. Effect sizes were reported as r for the Mann–Whitney U test and Hedges’s g for the independent-sample *t*-test. Higher IBS-QOL scores indicate poorer quality of life.

## Data Availability

The original contributions presented in this study are included in the article. Further inquiries can be directed to the corresponding author.

## Abbreviations

The following abbreviations are used in this manuscript:

IBS	Irritable Bowel Syndrome
FODMAP	Fermentable Oligosaccharides–Disaccharides–Monosaccharides and Polyols
ZFP	Zonulin Family Peptides
IBS-SSS	Irritable Bowel Syndrome–Symptom Severity Score
IBS-QOL	Irritable Bowel Syndrome–Quality of Life
IBS-D	Irritable Bowel Syndrome–Diarrhea-Predominant Type
IBS-C	Irritable Bowel Syndrome–Constipation-Predominant Type
IBS-M	Irritable Bowel Syndrome–Mixed Type
IBS-U	Irritable Bowel Syndrome–Unclassified Type
Tj	Tight Junction
LPS	Lipopolysaccharides
IL-6	Interleukin-6
IL-1β	Interleukin-1 Beta
TNF-α	Tumor Necrosis Factor Alpha
VAS	Visual Analog Scale
BMI	Body Mass Index
WBC	White Blood Cell
HGB	Hemoglobin
PLT	Platelet
AST	Aspartate Aminotransferase
ALT	Alanine Aminotransferase
CRP	C-Reactive Protein
FABP	Intestinal Fatty Acid-Binding Protein
DAO	Diamine Oxidase
